# Assessing the Giant Panda Protected Areas and Habitat Trends for Sympatric Endangered Species: A Climate Change Perspective

**DOI:** 10.1002/ece3.72179

**Published:** 2025-09-25

**Authors:** Yuanhao Du, Yuxin Jiang, Bingquan Zheng, Tianjiao Liu, Pei Yu, Mei Yang, Jianghong Ran

**Affiliations:** ^1^ Key Laboratory of Bio‐Resource and Eco‐Environment of Ministry of Education, College of Life Science Sichuan University Chengdu China; ^2^ The Conservation of Endangered Wildlife Key Laboratory of Sichuan Province Chengdu China; ^3^ Guidance and Service Center for the Development of Modern Forestry Industry in Liangshan Prefecture Xichang China

**Keywords:** climate change, concurrent trends, giant pandas, multi‐species conservation, protected area boundaries, sympatric endangered species

## Abstract

Climate change is a significant driver of biodiversity loss in the 21st century, primarily by affecting species distributions. We employed an optimized MaxEnt model to investigate concurrent habitat trends of giant pandas and sympatric endangered species—forest musk deer—under various climate scenarios in the Liangshan Mountains. Additionally, we assessed the effectiveness of protected area boundaries designed for flagship species. Our findings indicate that both species are projected to face different levels of habitat reduction, with declines ranging from 6.73% to 16.24% for giant pandas and from 1.53% to 24.17% for forest musk deer. Additionally, both species show a trend of migration towards the central areas and higher elevations in the Liangshan Mountains. The areas of jointly suitable habitat are also expected to decrease by 15.37%–37.15%, and the proportion of jointly suitable habitat within their respective suitable habitats will also diminish, highlighting both commonalities and differences in their responses to climate change. The overall habitat suitability for giant pandas is expected to improve, while the suitability for forest musk deer is expected to decline. However, habitat suitability within protected areas remains consistently higher than outside these zones, suggesting that the current boundaries of the protected areas are likely to remain effective in maintaining suitable environmental conditions for both species under future climate scenarios. We recommend developing joint conservation strategies for species inhabiting the Liangshan Mountains, where jointly suitable habitats are either stable or on the rise. These areas may serve as potential refuges for mammals with similar habitat preferences as climate change progresses. Our results also yield critical insights for biodiversity conservation in China and worldwide. They are especially pertinent in contexts where conservation frameworks prioritize a single flagship species, thereby overlooking the ecological requirements of sympatric yet equally endangered species.

## Graphical abstract

Here, we used a tuned MaxEnt model to investigate concurrent habitat trends of giant pandas and sympatric endangered species—forest musk deer—under various climate scenarios in the Liangshan Mountains. Additionally, we assessed the effectiveness of protected area boundaries designed for flagship species. The results show that both species show a trend of migration towards the central areas and higher elevations in the Liangshan Mountains, and the current range of the natural reserve is likely to remain effective in maintaining suitable environmental conditions for both species under future climate scenarios.

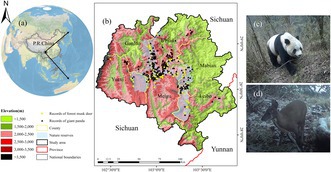

## 1 Introduction

Due to the pressures of global warming, approximately 50%–60% of all species worldwide are undergoing habitat loss and fragmentation (Koh et al. 2004; Bellard et al. 2012; Warren et al. 2013; Carthey and Blumstein 2018; Bongaarts 2019), causing significant declines in biodiversity (Sala et al. 2000; Travis 2003). For species unable to rapidly adapt their life history or physiological functions (Chen et al. 2011), the only remaining option may be to become “climate migrants”(Jonzén et al. 2006), relocating to higher altitudes or latitudes in search of suitable habitats based on their ecological preferences (Hannah et al. 2005; Jiang et al. 2020). Studies indicate that terrestrial species are moving upwards in elevations at a rate of 11 m per decade and shifting poleward at a rate of 16.9 km per decade (Chen et al. 2011), resulting in either habitat contraction or expansion (Parmesan and Yohe 2003). Consequently, understanding how climate change impacts species' habitats is crucial for the conservation of rare and endangered species.

Adopting a multi‐species perspective is critical for elucidating the complexity of ecological responses to climate change, particularly in regions where multiple endangered species coexist and may undergo coordinated or divergent shifts in habitat use (Bradshaw and Holzapfel 2006; Powers and Jetz 2019; Zhang et al. 2024). Previous studies have primarily focused on climate‐driven changes in species interactions—such as altered competition or predation—which have been shown to reshape the spatial distributions of sympatric species (Zhao et al. 2021; Dey et al. 2017; Stenseth et al. 2015; Wilson et al. 2022). However, these studies have largely overlooked the responses of sympatric endangered species. In addition, several studies have projected species distribution changes under climate change at broad taxonomic or geographic scales (Wu et al. 2017; Zhang et al. 2022). For instance, large‐scale assessments have forecasted distributional shifts for thousands of species under future climate scenarios. However, these studies tend to evaluate species either individually or through aggregated biodiversity metrics. As a result, a substantial gap remains in our understanding of how sympatric endangered species undergo divergent or synchronized habitat shifts. Given that conservation strategies are often implemented at the habitat or community scale, this gap constrains the development of spatially explicit and ecologically grounded in situ conservation plans.

Currently, most in situ conservation efforts globally are centered on a single “flagship” species (Smith et al. 2012; Verissimo et al. 2011), such as the giant panda, Ethiopian wolf ( *Canis simensis* ), West Indian manatee ( *Trichechus manatus manatus* ), and African elephant ( *Loxodonta africana* ; Caro and Riggio 2014; Duan and Yang 2020; Jaric et al. 2024; Mekonnen et al. 2024), which not only serve as symbols for conservation efforts but also attract significant funding (Shen et al. 2020). Flagship‐based strategies have, in some cases, achieved tangible conservation outcomes. For example, the wild population of the giant panda has increased from approximately 1100 individuals in the 1980s to nearly 1900 today. Habitat protection measures implemented for the panda have also delivered ancillary benefits to several sympatric species, including the golden snub‐nosed monkey and the snow leopard. Nevertheless, this approach has significant limitations. In Vietnam, the establishment of a national park to conserve the Javan rhinoceros ( *Rhinoceros sondaicus* ) failed to prevent the species' extinction in the wild in 2010 (Brook et al. 2014). Moreover, other sympatric species in the region, such as elephants and wild buffaloes, also suffered from inadequate protection. A key structural challenge of flagship‐oriented conservation is that protected areas are often delineated around the habitat of a single species, which involves complex and systematic planning, making them difficult to alter (Le Saout et al. 2013). Studies have found that only 9% of threatened species' habitats are effectively protected within these areas (Senior et al. 2024), and the effectiveness of these areas for protecting both flagship species and other sympatric endangered species remains underexplored under future climate conditions.

Traditional research of evaluating protected area effectiveness is often based solely on the overlap between species' current suitable habitats and the boundaries of protected areas (Li et al. 2016; Xu et al. 2014; Yang et al. 2021). However, emerging findings suggest that both habitat suitability inside and outside protected areas is crucial for species conservation, especially for mammalian habitats (Brodie et al. 2023). Evaluating effectiveness solely based on habitat suitability within protected areas overlooks developments outside these boundaries. To accurately assess the effectiveness of protected area boundaries under climate change, it is necessary to evaluate habitat changes both within and outside these areas and to understand shifting spatial patterns.

The giant panda ( *Ailuropoda melanoleuca* , VU; Figure S1) is a globally recognized endangered flagship species, coexisting with several other threatened species within its range, including the forest musk deer ( *Moschus berezovskii* , EN; Figure S2), Sichuan golden monkey ( *Rhinopithecus roxellana* , EN), snow leopard (*Panthera uncia*, VU), and Sichuan takin ( *Budorcas taxicolor* , VU; IUCN 2024). The forest musk deer, a representative species of the even‐toed ungulates, has experienced a severe population decline in China due to deforestation, habitat loss, and illegal hunting over recent decades (Yang et al. 2003; Yao et al. 2015). It is now classified as a National Class I protected wildlife species (National Forestry and Grassland Administration 2021) and listed as endangered (EN) by the IUCN in 2024. Even‐toed ungulates are particularly vulnerable to global warming due to their specialized food sources (Wu et al. 2017). Jiang et al. (2020) have found that the suitable habitat for six musk deer species in China will further shrink and shift towards higher latitudes over the next three decades. The Liangshan Mountains (LSM) represent the southernmost range of the giant panda, where it exists as a rear‐edge population (Li, Rao, et al. 2023). This area also contains six panda reserves (State Council Information Office of China 2013), as well as other rare and endangered species such as the red panda (*Ailurus styani*, EN), forest musk deer, and Chinese goral ( *Naemorhedus griseus* , VU; IUCN 2024). This study focuses on the giant panda and forest musk deer, two rare and endangered species with different phylogenetic backgrounds and seasonal migration patterns, to analyze trends in habitat suitability consistency and to examine changes in habitat suitability indices both inside and outside protected areas. The objective is to understand collective response strategies, habitat changes, shifts in habitat centers, and the effectiveness of protected area boundaries.

This study utilizes field survey data collected from 2019 to 2024 for both the forest musk deer and giant panda, as well as limited data from the Fourth National Panda Survey (2015). The MaxEnt model, combined with the latest climate data from the Coupled Model Intercomparison Project Phase 6 (CMIP6), was used to construct species distribution models for the LSM. The specific objectives are: (1) to predict the concurrent trends in suitable habitat changes for the forest musk deer and giant panda under climate change; (2) to explore shifts in the centers of suitable habitat for both species; and (3) to assess whether the LSM protected area cluster will meet the habitat needs of these rare and endangered species under future climate change. These results also provide reference points for biodiversity conservation and effectiveness assessments of protected areas in China as well as other regions throughout the world, especially in regions where conservation is centered around a single flagship species.

## 2 Methods

### 2.1 Study Area

**FIGURE 1:**
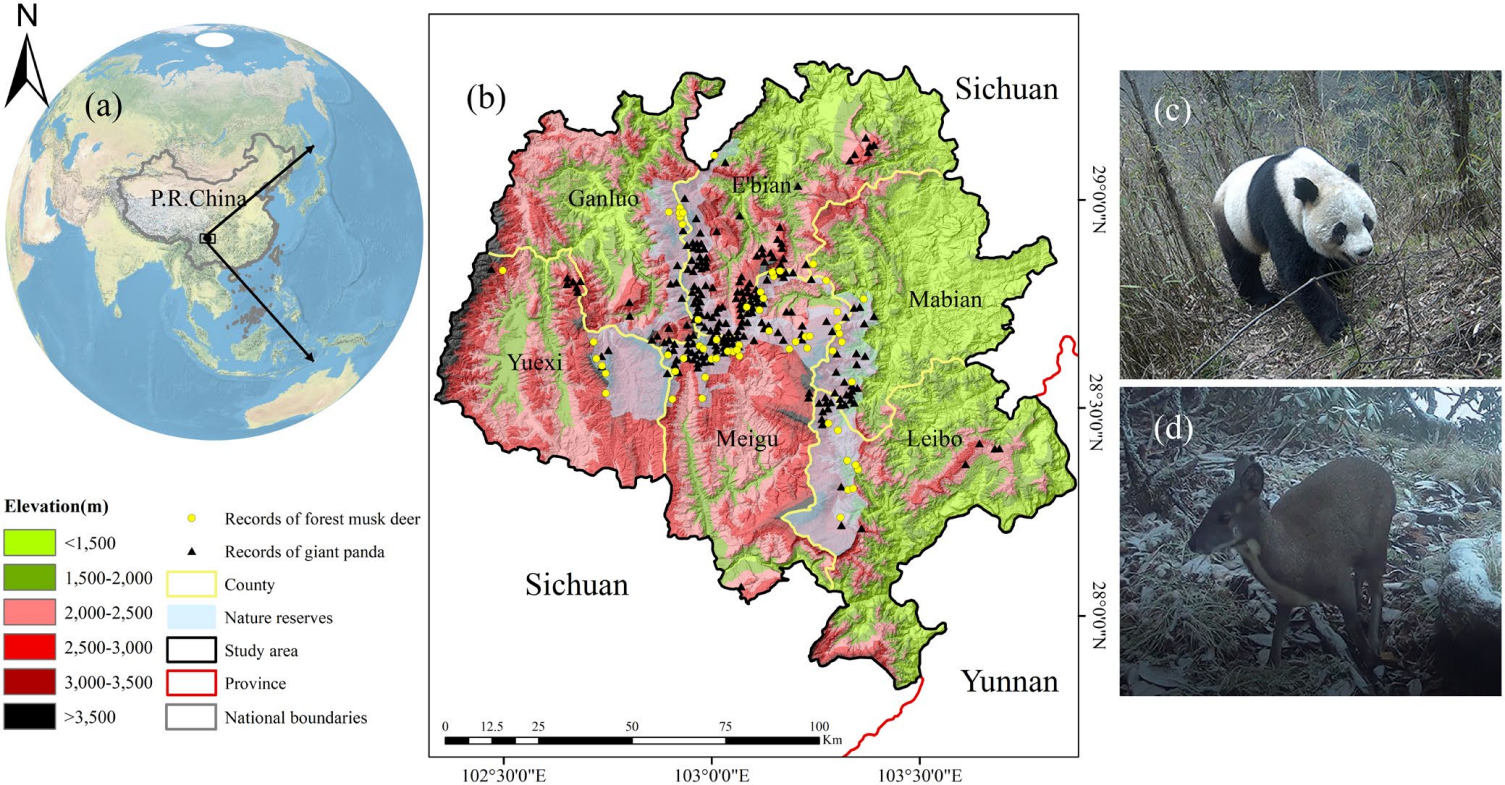
Map of the study area. 228 distribution points of giant panda and 60 distribution points of forest musk deer after filtering in the main ridge of Liangshan Mountains, which include six administrative counties.

The Liangshan Mountains are located at the transition between the Tibetan Plateau and the southwestern part of the Sichuan Basin. As one of the global biodiversity hotspots (Li et al. 2019), this region exhibits pronounced climatic heterogeneity and supports a high concentration of endemic species, making it particularly susceptible to disturbances from extreme climatic events (Tang et al. 2022). Situated at the southernmost edge of the six major mountain ranges of giant panda distribution, LSM hosts six contiguous giant panda reserves established to protect the species and its habitat. These reserves cover a total area of 1904.52 km^2^, with the elevation ranging from 310 m to 4288 m. The vegetation types within the reserves are diverse, including evergreen broadleaf forests, mixed coniferous and broadleaf forests, subalpine coniferous forests, alpine shrubs, and meadows. The study area encompasses the six reserves along the main ridge of the LSM and surrounding regions, covering an area of 14,653 km^2^, with coordinates ranging from 27°48′ N to 29°18′ N and 102°29′ E to 104°23′ E (Figure 1).

### 2.2 Data Sources

#### 2.2.1 Species Distribution Data

Distribution data for giant pandas and forest musk deer were obtained from field surveys conducted by our research team in the study area from 2019 to 2024. These surveys employed standardized protocols including transect walks, direct sightings, and sign surveys, conducted by trained field biologists. Infrared camera traps were also deployed at fixed monitoring stations in select reserves to supplement presence data. Additionally, a small account of distribution points for both species was obtained from the Fourth National Panda Survey (2015) to enhance the dataset. In total, 1867 giant panda distribution points were recorded, with 1535 located within the reserves and 332 outside. For forest musk deer, 121 distribution points were obtained, with 109 within the reserves and 12 outside. Each observation record contains precise GPS coordinates with an estimated spatial accuracy of ±10 m, along with the date and method of detection. Individuals were not marked or uniquely identifiable in this study, thus repeated observations of the same individual cannot be completely ruled out. Data collection followed standardized field protocols across all sites. All distribution points were validated by trained experts through direct observations, infrared camera footage, or verified species signs.

#### 2.2.2 Environmental Data

The accuracy of model predictions improves with the inclusion of various driving factors that influence species distribution (Li et al. 2022). Previous research indicates that habitat selection by giant pandas and forest musk deer is influenced by a combination of climate factors, vegetation types, food resources, topographical factors, and human disturbances.

##### 2.2.2.1 Climate Factors

Current climate scenarios are derived from WorldClim version 2.1 (https://worldclim.org/), providing 19 bioclimatic variables with a spatial resolution of 30 arc sec (1 km). Future climate variables are sourced from the Sixth Coupled Model Intercomparison Project (CMIP6), which offers projections based on different climate change and Shared Socioeconomic Pathways (SSPs). We used the medium‐resolution National (Beijing) Climate Center Climate System Model (BCC‐CSM2‐MR), which performs better in simulating key aspects such as tropospheric air temperature, regional circulation patterns across East Asia, and climate variability at different temporal scales (Wu et al. 2019). The same set of climate variables was selected for four time periods: 2021–2040 (2030s), 2041–2060 (2050s), 2061–2080 (2070s), 2081–2100 (2090s) of four Shared Socioeconomic Pathways (SSP1‐2.6, SSP2‐4.5, SSP3‐7.0, and SSP5‐8.5).

##### 2.2.2.2 Vegetation Types

LANDSAT remote sensing imagery (https://www.earthdata.nasa.gov/) with a spatial resolution of 30 m was downloaded. Preprocessing steps included radiometric calibration, atmospheric correction, and mosaicking, which were performed using ENVI. Supervised classification, based on field survey data and historical imagery from Google Earth Pro, categorized the vegetation into nine types: broadleaf forest, coniferous forest, mixed coniferous and broadleaf forest, bamboo forest, shrubland, meadow, cropland and residential areas.

##### 2.2.2.3 Source of Food

Forest musk deer primarily consume tender branches and leaves of grasses and plants (Guo 2023), while giant pandas rely mainly on bamboo (Wei et al. 2000). Thus, we used annual Net Primary Productivity (NPP) and ΔEnhanced Vegetation Index (EVI) obtained from the U.S. Geological Survey (https://lpdaac.usgs.gov/) to indicate the abundance of food resources in the region. NPP data from MOD17A3HGF (annual, 500 m resolution) were averaged for 2019–2024. EVI data from MODIS13Q1 (250 m resolution) were used to calculate annual EVI maximum and minimum values, and ΔEVI was derived using the raster calculator in ArcGIS 10.2.

##### 2.2.2.4 Topographical Factors

ASTER GDEM data (30 m resolution) from the China Geospatial Data Cloud (http://www.gscloud.cn/) were used to extract elevation, slope, and aspect layers in ArcGIS 10.2. Slope aspect was transformed from a circular to a continuous variable using the cosine function, with values near 1 indicating shadowed slopes and values near −1 indicating sunlit slopes. River vector layers from the National Geomatics Center of China (https://www.ngcc.cn/) were used to calculate Euclidean distances from each grid cell to the nearest river.

##### 2.2.2.5 Human Disturbance

Vector layers for roads and settlements were obtained from the National Geomatics Center of China. Euclidean distances from each grid cell to the nearest road and residential area were computed using ArcGIS 10.2. All 30 environmental variables were resampled to 30 m × 30 m grids, with continuous variables using bilinear interpolation and categorical variables using nearest neighbor assignment.

### 2.3 Data Analysis

#### 2.3.1 Habitat Model Selection and Optimization

The MaxEnt 3.4.1 model was used to simulate the potentially suitable habitats for the species (Phillips and Schapire 2019). To address model overfitting caused by excessive spatial autocorrelation among distribution points, redundant points within 1125 m for giant pandas and 610 m for forest musk deer were excluded based on their minimum home range areas of 3.9 and 1.17 km^2^, respectively (Hu and Schaller 1985; Liu et al. 2020). The preliminary models used 19 bioclimatic variables under current climate scenarios. Spearman correlation tests were conducted using IBM SPSS 25.0 software, and variables with high correlations (|*ρ*| > 0.8) were excluded to reduce collinearity and overfitting. Consequently, 15 environmental factors were retained for constructing species distribution models (Table 1). The regularization multiplier and combinations of features may impact the performance and accuracy of the model. The ENMeval 2.0.3 package in R was used to determine the optimal regularization multiplier and feature combinations (Kass et al. 2018). We evaluated five feature combinations: (1) L; (2) H; (3) LQ; (4) LQH; (5) LQHP, with regularization multipliers ranging from 0.5 to 5 in increments of 0.5. This process generated 60 models, with the model having the lowest Akaike Information Criterion (AIC) considered the best candidate (Warren and Seifert 2011). The optimal model for giant pandas used the LQ feature combination with a regularization multiplier of 0.5 (Appendix S1), whereas for forest musk deer, the optimal model also employed the LQ feature combination but with a multiplier of 1.5 (Appendix S2). The running parameters were set to randomly assign 75% of occurrence points to model training and 25% to testing, the output format was “Cloglog” (Phillips et al. 2017), the output file type was “ASCII,” the run type was set to “Bootstrap,” and the number of repetitions was 20. Variable importance was determined using the Jackknife test (Phillips et al. 2006). Model performance was evaluated using AUC and True Skill Statistic (TSS, Allouche et al. 2006), with the latter calculated in R using the SDMtune package (Vignali et al. 2020). TSS combines sensitivity and specificity and ranges from −1 to 1, with higher values indicating greater predictive accuracy (Allouche et al. 2006). Model overfitting was assessed by the test point omission rate based on the minimum training presence value (OR_MTP_), ranging from 0 (no overfitting) to 1.0 (overfitting; Peterson and Peterson 2011). For future climate scenarios, all non‐climatic predictors were kept identical to the current climate models, using the same parameter settings.

**TABLE 1:** Species distribution model variables.

	Abbreviation	Description	Original resolution	Species
Climatic factors	Bio1	Annual mean temperature	1 km	All
	Bio2	Mean diurnal range	1 km	All
	Bio7	Temperature annual range	1 km	Forest musk deer
	Bio12	Annual precipitation	1 km	All
	Bio13	Precipitation of wettest month	1 km	All
	Bio14	Precipitation of driest month	1 km	Giant panda
	Bio15	Precipitation seasonality	1 km	Forest musk deer
	Bio16	Precipitation of wettest quarter	1 km	Giant panda
Vegetation factors	Vegetation	Vegetation type	30 m	All
Source of food	Npp	Net primary production	500 m	All
	ΔEVI	ΔEnhanced vegetation index	250 m	All
Topographical factors	Elevation	Altitude	30 m	All
	Aspect	Aspect	30 m	All
	Slope	Slope	30 m	All
	Dist_river	Distance from rivers	Extract	All
Human disturbance	Dist_settlement	Distance from settlements	Extract	All
	Dist_road	Distance from roads	Extract	All

#### 2.3.2 Habitat Analysis

##### 2.3.2.1 Overlap of Suitable Habitats for Both Species

To determine the thresholds for suitable and unsuitable habitats under both current and future climate scenarios, we used the maximum test sensitivity plus specificity from the model outputs. This method was selected because it is unaffected by the proportion of known presences and random background samples (Liu et al. 2013), and habitats with a Habitat Suitability Index (HSI) greater than this threshold were considered suitable for the species. Using ArcGIS 10.2, the suitable habitat distributions for both species under current and future climate scenarios were identified. The Raster Calculator tool was utilized to multiply the current predicted suitable habitat layers for the two species by the corresponding time‐period layers for each climate scenario, resulting in 16 change layers representing the variation in habitat distribution compared to the current conditions. The ENMTools 1.4.4 package in R was utilized to calculate the ecological niche overlap between giant pandas and forest musk deer under the current climate scenario. The niche overlap indices, Schoener's D (*D*) and Hellinger's‐based I (*I*), range from 0 to 1, where 0 indicates no ecological niche overlap and 1 indicates complete overlap of habitats suitable for both species (Warren et al. 2010).

Shifts in the habitat barycenter under future scenarios can reveal directional trends in range dynamics, providing spatial guidance for adaptive conservation planning. The longitude and latitude of the study area were divided into 0.1° × 0.1° grid cells to calculate the barycenter of the jointly suitable habitat for giant pandas and forest musk deer under future climate scenarios. Assuming that the study area consists of *n* sub‐primary regions *i*, *M*_*i*_ represents the magnitude of the *i*th sub‐primary region and is calculated by the following equation: *M*_*i*_ = *ρ*_*i*_ × *S*_*i*_. Here, *ρ*_*i*_ represents the probability of presence of the species, which was obtained by extracting the property values of each cell, and *S*_*i*_ represents the area of the *i*th region, based on the number of grid cells. Then, (*X*_*i*_, *Y*_*i*_) represents the longitude and latitude of the *i*th sub‐primary central region, respectively (Jiang et al. 2020). The barycenter of this region can be expressed by the following equations:

$$X=\frac{\sum_{i=1}^{n}M_{i}X_{i}}{\sum_{i=1}^{n}M_{i}},Y=\frac{\sum_{i=1}^{n}M_{i}Y_{i}}{\sum_{i=1}^{n}M_{i}}$$

#### 2.3.3 Changes in Habitat Suitability Index (HSI)

First, we computed the difference in the Habitat Suitability Index (HSI) for each grid cell within the study area under future climate scenarios compared to the current climate scenario. To evaluate whether the giant panda nature reserve system adequately covered the conservation needs for giant panda and sympatric species, we used the maximum value of the standard deviation of species HSI variation among different model runs as the threshold (Wang et al. 2021), and categorized species habitat change into “improved” (ΔHSI > 0.1), “stable” (−0.1 > ΔHSI > 0.1), and “decreased” (ΔHSI < −0.1). We then compared species habitat change in and outside nature reserves.

## 3 Results

### 3.1 Habitat Selection and Model Performance Under Current Climate Scenario

**FIGURE 2:**
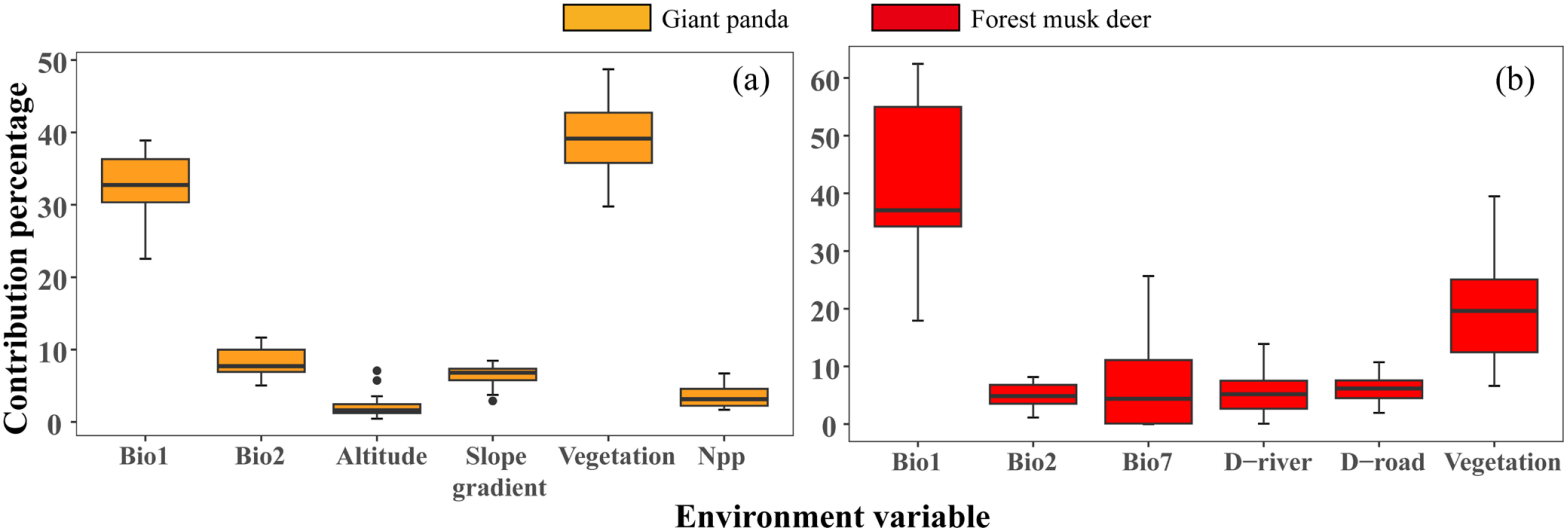
Mean contribution of the six most important environmental variables in predicting the potential distributions of giant panda and forest musk deer under the current period. (a) Giant panda; (b) Forest musk deer; Bio1: Annual Mean Temperature; Bio2: Mean Diurnal Range; Bio7: Temperature Annual Range; NPP: Net Primary Production; D‐river: Distance from river; D‐road: Distance from road; Vegetation: Vegetation types.

**FIGURE 3:**
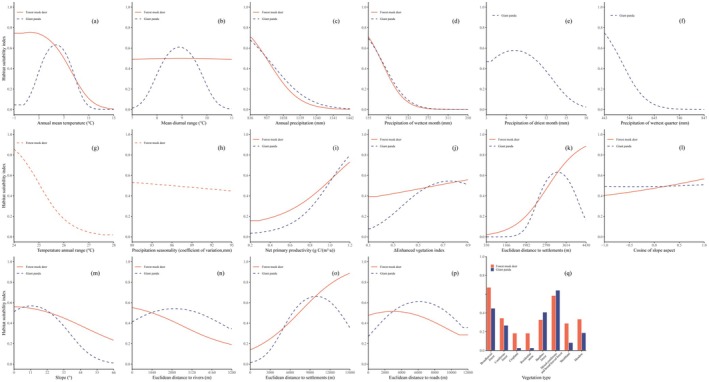
Response curves of environmental variables under the current climate scenario. The red solid line represents the species forest musk deer, while the blue dashed line represents the giant panda.

After spatial screening, a total of 228 giant panda and 60 forest musk deer occurrence points were retained for habitat modeling. Through 20 repetitions of cross‐validation, the mean training AUC, mean test AUC, maximum TSS, and OR_MTP_ values were 0.94, 0.93, 0.79, and 0.02 for giant pandas, and 0.93, 0.88, 0.75, and 0.09 for forest musk deer, respectively. These results indicate that the MaxEnt models showed high fit and effectively simulated the spatial distributions of both species in the LSM. Variable importance analysis revealed that vegetation type (39.4%) was the most influential factor for giant panda habitat modeling, followed by mean annual temperature (bio‐1p; 32.6%), mean diurnal range (bio‐2p; 8.1%), and slope (6.5%; Figure 2a). For forest musk deer, the most influential factors were mean annual temperature (bio‐1M; 41.7%), vegetation type (19.3%), temperature annual range (bio‐7M; 7.3%), and distance to roads (D‐road; 6.2%; Figure 2b). The cumulative contribution rates of climate factors in the models were 43.5% for giant pandas and 55.5% for forest musk deer. Habitat suitability evaluations revealed both similarities and differences in the environmental factors affecting the distributions of giant pandas and forest musk deer. For example, both species preferred habitats with coniferous forests and mixed coniferous‐deciduous forests, as well as a mean diurnal range of 9°C. Additionally, giant pandas preferred bamboo forests, while forest musk deer preferred broadleaf forests (Figure 3).

According to MaxEnt model predictions under the current climate scenario, suitable habitat areas for giant pandas and forest musk deer in the LSM are 2009.88 km^2^ and 2744.80 km^2^, respectively, with a jointly suitable habitat area of 1644.96 km^2^ (Table 2). The jointly suitable habitat for both species is mainly concentrated within the protected areas, comprising 59.06% of the total jointly suitable habitat, with a slight distribution outside the protected areas also occurring in the transition zones between E'bian County and Mabian County, as well as in the northern part of Leibo County (Figure S3). Niche overlap tests for giant pandas and forest musk deer under the current climate scenario revealed low ecological niche overlap (*D* = 0.20 and *I* = 0.29), indicating that the two species can achieve stable coexistence.

**TABLE 2:** Suitable habitat areas for giant pandas and forest musk deer in the LSM under current climate scenario.

Species	Liangshan mountains (km^2^)			In the nature reserve (km^2^)			Outside the nature reserve (km^2^)		
	Suitable habitat	Unsuitable habitat	Common suitable habitat	Suitable habitat	Unsuitable habitat	Common suitable habitat	Suitable habitat	Unsuitable habitat	Common suitable habitat
Giant pandas	2009.35	12,404.85	1644.96	1026.53	1103.54	971.54	982.82	11,301.31	673.42
Forest musk deer	2744.80	11,535.98		1493.14	629.00		1251.66	10,906.98	

### 3.2 Changes in Suitable Habitat of Giant Pandas and Forest Musk Deer Under Climate Change

**FIGURE 4:**
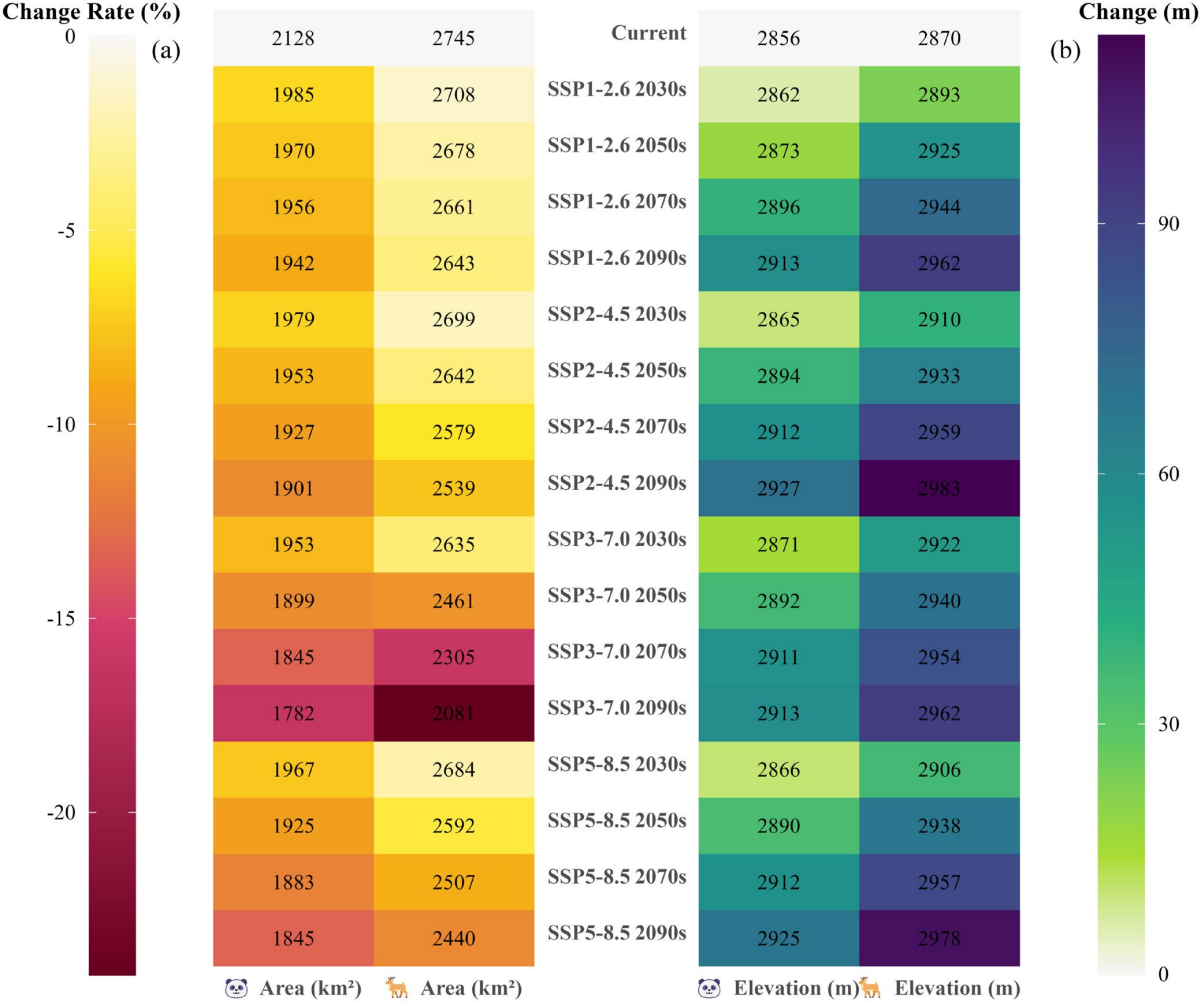
Projected changes in habitat area and mean elevation for the giant panda and forest musk deer under different climate change scenarios. In panel (a), each cell displays the absolute area of suitable habitat (km^2^), while in panel (b), the values indicate the mean elevation (m) of suitable habitat. Positive values (shades of blue) indicate an increase in mean elevation, with darker shades corresponding to larger elevational shifts. Negative values (shades of red) indicate a reduction in suitable habitat area relative to the current distribution, with darker shades reflecting greater habitat loss.

The models predicted changes in suitable habitat for giant pandas and forest musk deer in the LSM under four different climate scenarios. Compared to the current climate scenario, the suitable habitat for both species is projected to shrink under future climate scenarios. Under the SSP3‐7.0 scenario, giant pandas face the most severe habitat loss by the 2090s, with only 1782.35 km^2^ of suitable habitat remaining (reduced by 16.24%). Whereas the smallest reduction occurs by the 2030s under the SSP1‐2.6 scenario, leaving 1984.70 km^2^ (reduced by 6.73%; Figure 4a). For forest musk deer, the smallest suitable habitat area is projected to be during the 2090s under the SSP3‐7.0 scenario, with a loss of 663.44 km^2^, leaving only 2081.36 km^2^ (reduced by 24.17%). Conversely, under the SSP1‐2.6 scenario, the largest suitable habitat area is expected by the 2030s, with 2707.67 km^2^ remaining, losing only 37.13 km^2^ (reduced by 1.35%; Figure 4a). In terms of vertical distribution, the average elevation of suitable habitat for both species under all climate scenarios is projected to be significantly higher than the current average elevation. Specifically, under the SSP3‐7.0 scenario, the average elevation of forest musk deer habitat will rise to 2962 m by the end of the century, approximately 92 m higher than the current average elevation. Similarly, the average elevation of giant panda habitat will increase to 2913 m by the end of the century, about 57 m higher than the current average elevation (Figure 4b).

**FIGURE 5:**
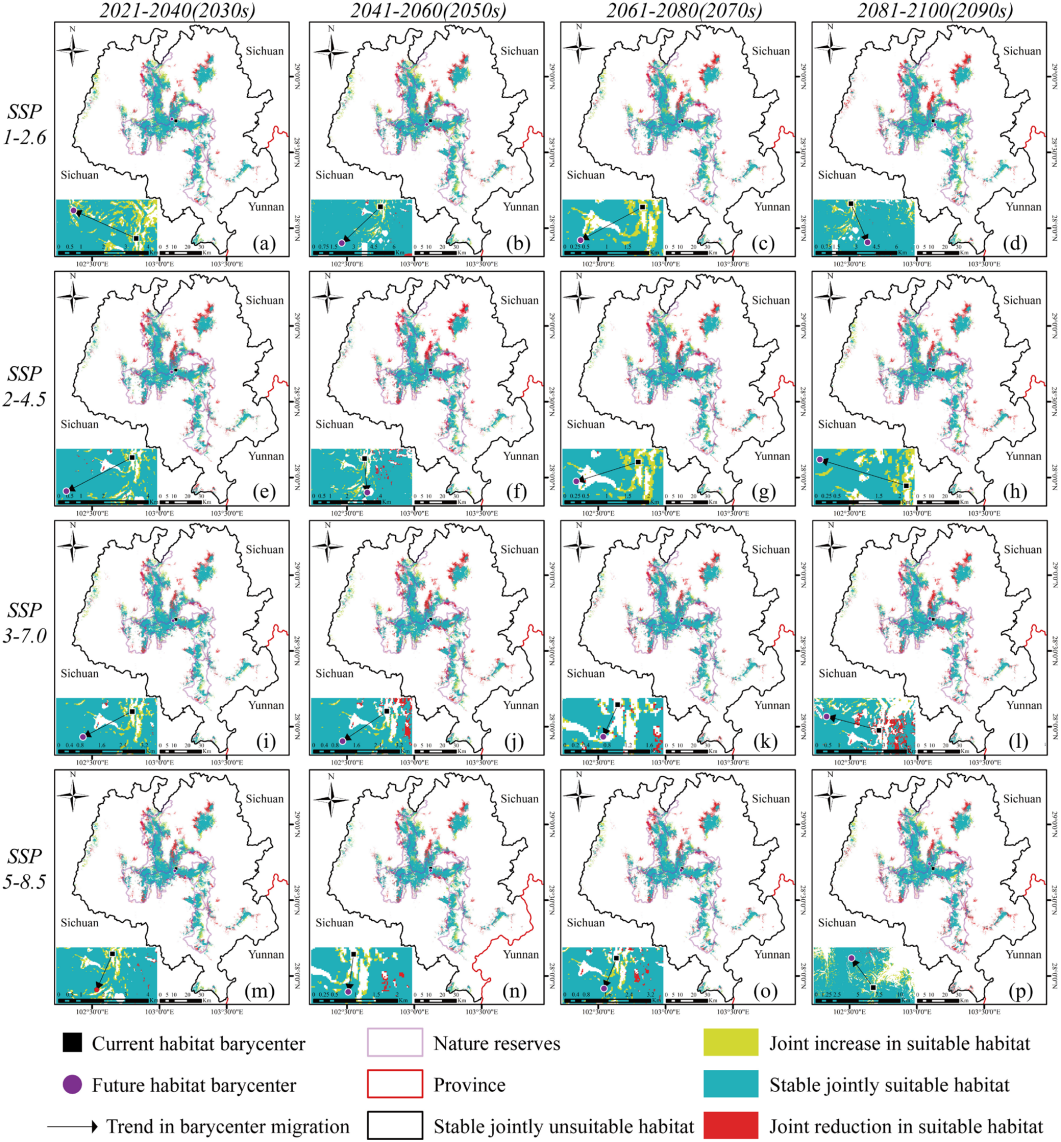
Trends in the jointly potential suitable habitats of giant panda and forest musk deer under climate change of shared socio‐economic pathway 1–2.6 (top), 2–4.5 (second row), 3–7.0 (third row), 5–8.5 (bottom) during different periods of the 21st century: (a, e, i, m) 2020–2040, (b, f, j, n) 2040–2060, (c, j, k, o) 2060–2080, (d, h, l, p) 2080–2100. The lower left corner of the small figure indicates the migration of the barycenter of jointly suitable habitat from the current climate scenario to this period, and all barycenters remain within the protected areas under climate change.

Across the four climate scenarios, the jointly suitable habitat for giant pandas and forest musk deer in the LSM shows a decreasing trend over time, with significant changes in horizontal distribution (Figure 5). The expansion of jointly suitable habitat is predominantly concentrated in the central and northern regions of the LSM, with the largest increase of 262.67 km^2^ occurring under the SSP1‐2.6 scenario in the 2030s. The contraction of jointly suitable habitat is mainly observed in E'bian County, with the greatest reduction of 389.83 km^2^ occurring under the SSP3‐7.0 scenario in the 2090s. Clearly, the area of habitat contraction always exceeds the area of habitat expansion. The direction of barycenter shift for the jointly suitable habitat of giant pandas and forest musk deer under climate change is relatively dispersed, predominantly moving towards the southwest. However, all barycenters remain within the protected areas. The distance of barycenter movement varies by period, with an average migration distance of 2.24 km. The farthest centroid shift is 4.24 km in the 2090s under the SSP5‐8.5 scenario, while the nearest shift is 0.65 km in the 2070s under the SSP3‐7.0 scenario (Figure 5).

### 3.3 Changes in Habitat Suitability Index (HSI) for Giant Pandas and Forest Musk Deer Under Climate Change

**FIGURE 6:**
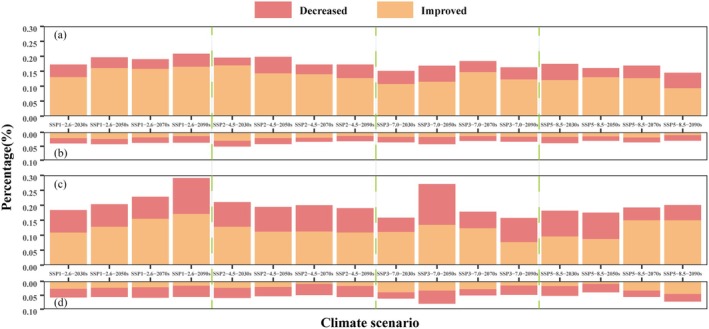
Degree of variation in habitat suitability within and outside reserves. Decreased represents ΔHSI < −0.1 and improved represents ΔHSI > 0.1. (a) giant panda within reserves; (b) giant panda outside reserves; (c) forest musk deer within reserves; (d) forest musk deer outside reserves.

Under various climate scenarios, the entire LSM region shows the net improvements in habitat for giant pandas (percentage of grids with ΔHSI > 0.1 minus percentage of grids with ΔHSI < −0.1 is greater than 0; Figure 6a,b). When comparing changes in HSI inside and outside the protected areas, the protected areas consistently show a net increase in habitat suitability, while outside the protected areas, net habitat increase occurs only under SSP1‐2.6 scenarios for the 2030s and 2050s, and SSP2‐4.5 scenarios for the 2030s and 2070s (0.21%, 0.56%, 1%, 0.51%), with rates significantly lower than those inside the protected areas (8.83%, 12.16%, 14.38%, 10.76%; Figure 6a,b). The remaining periods outside the protected areas show a net loss in habitat (percentage of grids with ΔHSI > 0.1 minus percentage of grids with ΔHSI < −0.1 is less than 0). In contrast, forest musk deer are more susceptible to climate change, with the net habitat loss across the entire LSM range (Figure 6c,d). Notably, within the protected areas, forest musk deer generally exhibit the net improvements in habitat, except for the 2050s and 2090s under SSP3‐7.0 scenarios and the 2050s under SSP5‐8.5 scenario, which show the net habitat loss (0.16%, 0.39%, 0.06%). However, the net habitat loss rates outside the protected areas during the same periods (1.36%, 1.78%, 2.07%) are higher than those inside the protected areas, with the remaining periods inside the protected areas also showing better changes compared to outside. Overall, regardless of the climate scenario, habitat suitability for both giant pandas and forest musk deer is better preserved within the protected areas than outside.

## 4 Discussion

### 4.1 Concurrent Trends in Suitable Habitat Under Climate Change

Accurately predicting the concurrent trends in suitable habitats for sympatric endangered species and assessing the effectiveness of protected area boundaries under climate change is crucial for guiding conservation strategies (Duffield et al. 2021; Turner et al. 2009). Our models assume static human settlements and road networks, but human disturbance may intensify with ongoing urban expansion and land‐use change. Although future projections of anthropogenic pressure are lacking, incorporating such dynamics in future models is essential. Areas near urban centers—such as eastern LSM—may face greater risks and should be prioritized for monitoring and adaptive management. Additionally, the limited presence data, particularly for forest musk deer, may reduce model accuracy in undersampled regions. Expanding field surveys would improve model reliability and help validate projected range shifts under climate change.

Our study reveals that climate factors contribute 43.2% and 55.5% to the habitat selection of giant pandas and forest musk deer. This indicates that climate factors are primary determinants of habitat selection for both species. The high overlap in habitats between giant pandas and forest musk deer in the LSM is evident, though the low niche overlap index suggests they occupy different microhabitats (Wei et al. 2000). For sympatric species to coexist harmoniously in concurrent habitats, they must exhibit niche differentiation in at least one spatial dimension such as dietary preferences, foraging sites, or microhabitat selection (Zhang et al. 2004). For instance, forest musk deer primarily consume tender shoots of grasses and herbaceous plants and prefer steep‐sloped coniferous and bamboo forests (Jian et al. 2001; Lvbing et al. 2008), whereas giant pandas favor gently sloped coniferous forests and bamboo forests, mainly feeding on bamboo (Wei et al. 2000). These differing niches facilitate their coexistence (Figures 3 and S3).

Previous studies have shown that in response to global warming, many species are migrating to higher elevations or latitudes (Chen et al. 2011; Parmesan and Yohe 2003). Our findings support this trend, with rising habitat elevations potentially lowering human disturbance and promoting population persistence (Figure 4). The observed southwestward shift in the barycenter of jointly suitable habitat likely reflects the combined influence of climatic and topographic factors. Notably, the southwestern portion of the LSM contains a higher proportion of high‐elevation terrain, offering cooler microclimates and greater topographic heterogeneity, which may enhance climatic buffering (Figure 1). However, it is important to note that high‐altitude areas in the LSM are both rugged and steep (Li et al. 2019), which could require significant energy expenditure for giant pandas and forest musk deer to search for food or mates. Additionally, higher elevations are associated with food scarcity because plant dispersal capabilities are limited, and new potential habitats may not be colonized rapidly without human intervention to facilitate their spread (Li et al. 2015a). Our research indicates a gradual reduction in the jointly suitable habitats for giant pandas and forest musk deer under climate change, with a decrease ranging from 15.37% to 37.15% (Figure 5). The proportion of jointly suitable habitats within each species' range also continues to decline. The differences in habitat changes between the two species may be attributed to their varying sensitivities or adaptability to climate change (Butt et al. 2016). Furthermore, the expansion of jointly suitable habitats is predominantly concentrated in the central and northern regions of the LSM (Figure 5), and this area may potentially serve as a refuge for species with similar habitat preferences to giant pandas or forest musk deer under future climate scenarios (Keppel and Wardell‐Johnson 2012).

### 4.2 Effectiveness of Protected Area Boundaries Under Climate Change

While the network of protected areas is essential for mitigating biodiversity loss, their long‐term effectiveness cannot be ensured by designation alone. Many suffer from limited management capacity, and the compounded effects of climate change and habitat loss may erode their conservation potential (Asamoah et al. 2021). Recent assessments of protected area effectiveness under future climate scenarios have yielded mixed outcomes (Brodie et al. 2023). For instance, Rubidge et al. (2024) found that suitable habitats for 34 benthic fish species in the Canadian Arctic continental shelf are likely to remain within current protected areas under all projected climate scenarios. In contrast, Leao et al. (2023) reported that although 16 carnivore species occur within Amazonian reserves, these reserves fail to effectively safeguard species richness across all climate scenarios, as null model analyses indicate. Our study reveals that under climate change, the proportion of increased HSI for giant pandas within protected areas significantly exceeds that of decreased HSI, reflecting a net gain (Figure 6). This finding is consistent with Wang et al.'s (2021) observations of improved HSI for giant pandas in the Qinling region over the past decade, and Tang et al. (2023) prediction that the panda reserve system will effectively protect giant pandas under intensified global warming. However, it contrasts with previous concerns that static reserve boundaries may be inadequate for long‐term species conservation under changing climatic conditions (Li et al. 2015a, 2017). Ultimately, conservation outcomes depend not only on spatial coverage but also on how protected areas are managed. Long‐term conservation strategies implemented by the Chinese government appear to have contributed to population recovery, as evidenced by the downlisting of the giant panda from Endangered to Vulnerable (Wei et al. 2015; The State Council Information Office 2021). Furthermore, the study found that habitat quality for black bears and forest musk deer has improved more significantly outside the Qinling reserves within a 20 km radius compared to inside (Wang et al. 2021). This pattern underscores the importance of incorporating surrounding landscapes into conservation planning, while also revealing a critical limitation of current protected area boundaries. Static reserve delineations may fail to capture the most ecologically favorable areas under shifting climate conditions, reinforcing the need for flexible, landscape‐scale strategies that extend beyond existing boundaries.

**FIGURE 7:**
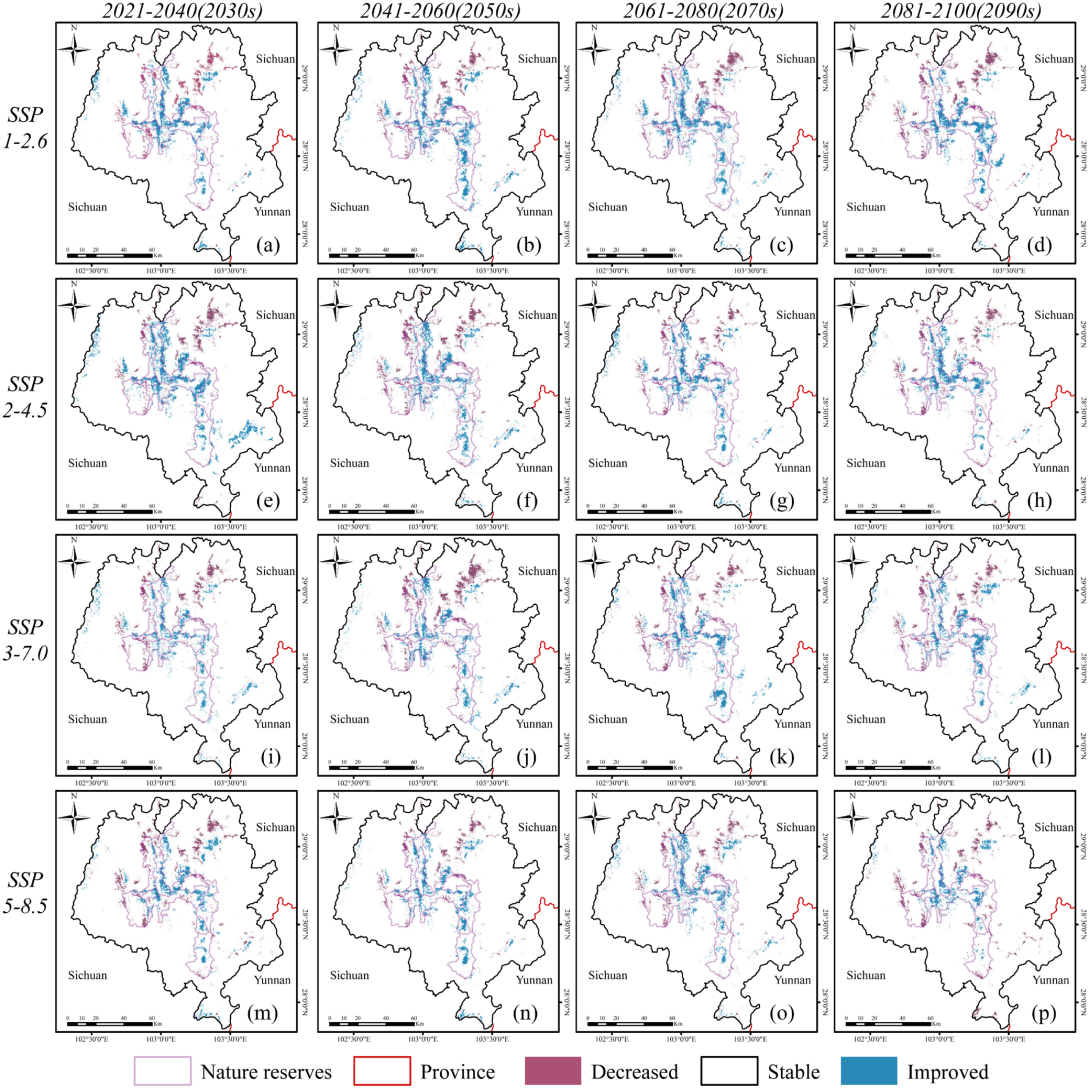
Variation in habitat suitability for giant panda under climate change of shared socio‐economic pathway 1–2.6 (top), 2–4.5 (second row), 3–7.0 (third row), 5–8.5 (bottom row) during different periods of the 21st century: (a, e, i, m) 2020–2040, (b, f, j, n) 2040–2060, (c, j, k, o) 2060–2080, (d, h, l, p) 2080–2100. Decreased: ΔHSI < −0.1; Stable: −0.1 > ΔHSI > 0.1; Improved: ΔHSI > 0.1.

The observed increase in HSI within the protected areas is characterized by a gradient from the edges towards the core (Figure 7). This change is likely attributable to China's implementation of the Natural.

Forest Protection Program (NFPP) coupled with the Grain‐for‐Green policy (GTG) over the past 20 years, which has led to a complete ban on logging, resulting in expanded forest cover and reduced non‐forest areas (Central Committee of the Communist Party of China 2019). This has mitigated the impact of climate change, as the contribution of vegetation type to the model's accuracy has increased, and giant pandas continue to prefer bamboo forests and mixed coniferous‐broadleaf forests. However, areas with “decreased” HSI are distributed across six protected areas (Figure 7), indicating that climate change exacerbates habitat fragmentation for giant pandas (Zang et al. 2020), potentially reducing genetic exchange between populations and thereby decreasing genetic diversity and survival potential (Li, Zhou, et al. 2023). The southeastern part of E'bian County, which is the region most severely impacted by climate change outside the protected areas (Figure 7), is also identified in the fourth panda survey (2015) as part of the giant panda distribution area and has the widest distribution of giant pandas in the LSM. Given the giant panda's specific habitat requirements such as lower slopes, abundant bamboo, and minimal human disturbance (Li et al. 2015b), it is crucial to implement active habitat restoration measures, develop regional conservation strategies, and establish migration corridors to address climate change and facilitate the population's expansion into core protected areas.

Under future climate scenarios, the HSI for forest musk deer in the LSM appears less promising compared to that of giant pandas, with an overall trend of net habitat loss. The least loss is projected under the SSP1‐2.6 scenario, while the most severe loss occurs under the SSP5‐8.5 scenario (Figure 6). Over recent decades, the population of forest musk deer in China has declined from approximately 1,000,000 to around 31,800 individuals (Jiang 2021), but the establishment of protected areas seems to be gradually reversing this trend. For instance, after the establishment of the Tangjiahe Nature Reserve, which primarily focuses on giant pandas, the forest musk deer population density remained relatively stable over six years (Yang et al. 2003). Similarly, ungulates such as the Chinese goral and the Chinese serow have received effective protection in the Baishuijiang Nature Reserve (Rong et al. 2019).

**FIGURE 8:**
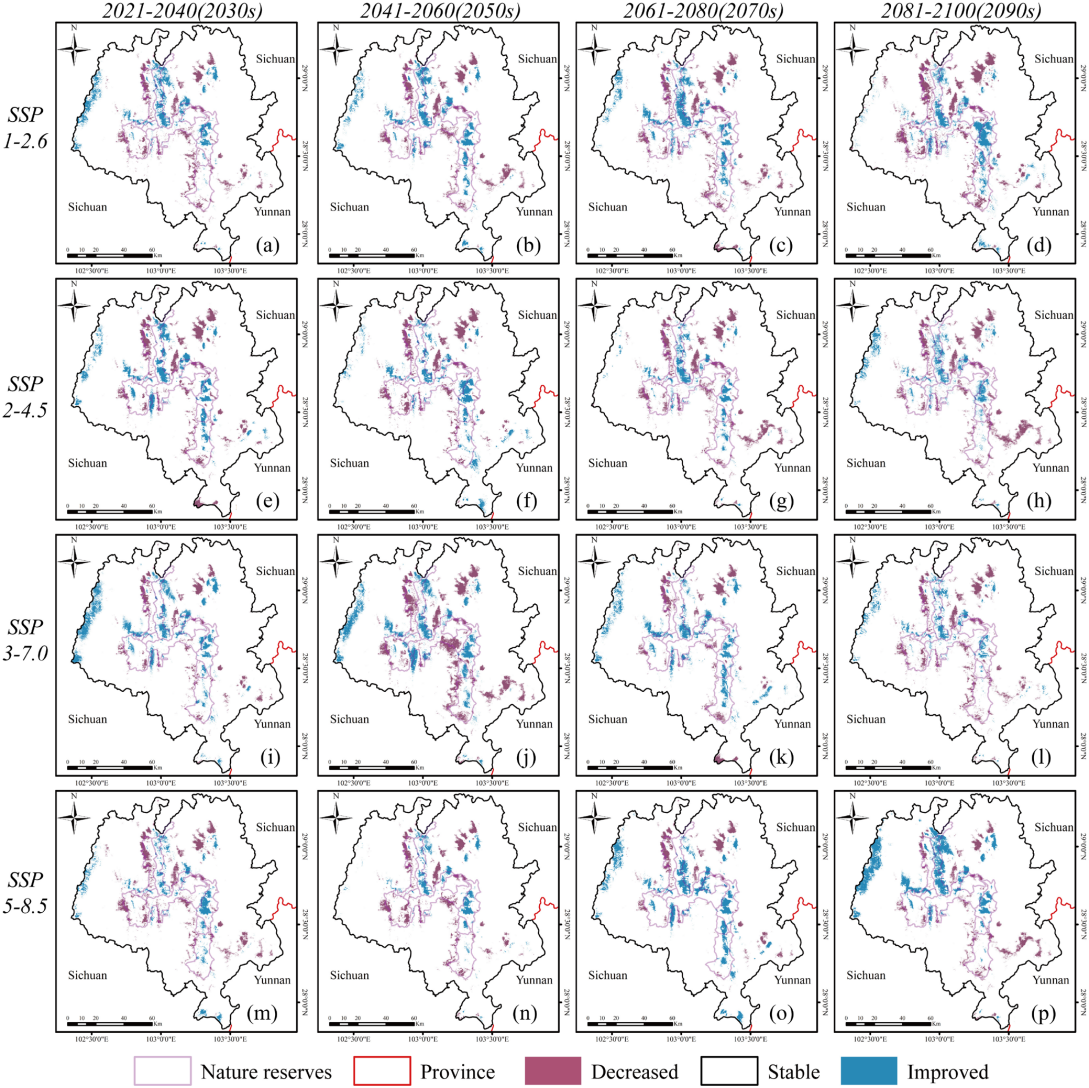
Variation in habitat suitability for forest musk deer under climate change of shared socio‐economic pathway 1–2.6 (top), 2–4.5 (second row), 3–7.0 (third row), 5–8.5 (bottom row) during different periods of the 21st century: (a, e, i, m) 2020–2040, (b, f, j, n) 2040–2060, (c, j, k, o) 2060–2080, (d, h, l, p) 2080–2100. Decreased: ΔHSI < −0.1; Stable: −0.1 > ΔHSI > 0.1; Improved: ΔHSI > 0.1.

Our results indirectly support these observations, as HSI changes within protected areas consistently outperform those outside the reserves. Even when HSI shows net habitat loss within the protected areas, the degree of loss is less severe compared to outside the reserves. Thus, our findings suggest that the current boundaries of the LSM protected area network are likely to remain effective in maintaining suitable environmental conditions for both giant pandas and forest musk deer under future climate scenarios. In this study, “effectiveness of protected area network” refers specifically to the relative maintenance or improvement of habitat suitability under projected climate conditions, and does not encompass broader ecological factors such as population size, spatial connectivity, genetic exchange, or demographic viability. As noted earlier, habitat fragmentation and potential limitations on gene flow remain substantial concerns. To more comprehensively evaluate the long‐term conservation value of these protected areas, future studies should incorporate population viability analyses and long‐term demographic monitoring under changing environmental conditions. Within the protected areas, the increase in HSI is primarily concentrated in the eastern and western regions (Figure 8). These areas may serve as future refuges for ungulates in the LSM. However, similar to giant pandas, the decline in HSI for forest musk deer underscores the need for adjustments in management policies to address climate change threats.

Notably, HSIs outside the protected areas are primarily found in the western part of LSM (in Ganluo County and Yuexi County, Figure 8), where forest musk deer are still present but are located far from the protected areas. Yao et al. (2015) found that the decline in forest musk deer populations in southwestern China may be attributed to poaching.

## 5 Conclusions and Conservation Recommendations

### 5.1 Conclusions

This study utilized the MaxEnt model to predict changes in suitable habitats for giant pandas and forest musk deer under climate change, revealing the similarities and differences in how these species respond to shifts in habitat suitability. By comparing changes in the HSI both within and outside protected areas, we assessed whether the LSM protected area network can meet the habitat needs of these rare and endangered species under future climate scenarios. The results indicate that both giant pandas and forest musk deer habitats are projected to diminish to varying degrees under future climate scenarios, with a trend towards migration to the central and higher elevation regions of LSM (decreasing by 6.73%–16.24% for giant pandas and 1.53%–24.17% for forest musk deer). This likely represents a shared strategy for adapting to climate change and underscores the urgency and importance of implementing targeted conservation policies to sustain mammalian habitats in LSM. Unfortunately, much of the LSM remains outside the boundaries of the Giant Panda National Park. The area of jointly suitable habitat for giant pandas and forest musk deer also shrinks (decreasing by 15.37%–37.15%), with the proportion of joint habitat within each species' suitable habitat gradually decreasing, suggesting differential habitat changes for the two species under climate change.

Additionally, future research should delve deeper into the specific environmental factors causing the differences in habitat suitability changes for these two species under climate change. Under future climate scenarios, the HSI for giant pandas in LSM shows the net improvements in suitable habitat, with this increase concentrated within the protected areas and significantly exceeding changes observed outside the protected areas. Conversely, although the HSI for forest musk deer consistently shows net habitat loss with climate change, changes within protected areas are still more favorable than those outside. Existing protected areas encompass most of the regions with increased net habitat for both giant pandas and forest musk deer, suggesting that the current boundaries of the protected areas are likely to remain effective in maintaining suitable environmental conditions for both species under future climate scenarios.

### 5.2 Conservation Recommendations

- Establish ecological corridors. Connect areas experiencing projected declines in habitat suitability with core protected regions to promote species dispersal and maintain genetic connectivity.
- Strengthen protection in the central and northern LSM. Prioritize conservation efforts in these regions where shared suitable habitats are expected to remain stable or expand. Implement joint‐species strategies through coordinated management, monitoring, and control of human disturbances such as grazing and bamboo harvesting.
- Reinforce anti‐poaching efforts outside reserves. In addition to regular patrols, targeted measures should include establishing community‐based wildlife monitoring networks, providing conservation‐linked livelihood incentives, and strengthening enforcement capacity in identified poaching hotspots.

## Author Contributions

**Yuanhao Du:** conceptualization (equal), data curation (equal), formal analysis (equal), investigation (equal), methodology (equal), visualization (equal), writing – original draft (equal), writing – review and editing (equal). **Yuxin Jiang:** conceptualization (equal), methodology (equal). **Bingquan Zheng:** conceptualization (equal), methodology (equal), writing – review and editing (equal). **Tianjiao Liu:** conceptualization (equal), methodology (equal). **Pei Yu:** writing – review and editing (equal). **Mei Yang:** investigation (equal). **Jianghong Ran:** conceptualization (equal), methodology (equal), supervision (equal), writing – review and editing (equal).

## Conflicts of Interest

The authors declare no conflicts of interest.

## Supporting information


**Figure S1:** Geographic distribution of the giant panda ( *Ailuropoda melanoleuca* ; IUCN 2024).

**Figure S2:** Geographic distribution of the forest musk deer ( *Moschus berezovskii* ; IUCN 2024).

**Figure S3:** Potential suitable habitats for giant panda and forest musk deer under the current period. The suitable habitat area solely for forest musk deer is 1099.84 km^2^, while that for giant panda is 364.39 km^2^, and the jointly suitable habitat area for both species is 1644.96 km^2^.

**Appendix S1:** Selection of feature combination and regularization multiplier for optimizing MaxEnt model complexity of giant pandas.

**Appendix S2:** Selection of feature combination and regularization multiplier for optimizing MaxEnt model complexity of forest musk deer.

## Data Availability

All the required data is uploaded as Figures [Link], [Link] and Appendices [Link], [Link].
