# Supplementary figures and images for: Assessing the Giant Panda Protected Areas and Habitat Trends for Sympatric Endangered Species: A Climate Change Perspective
Source: Ecol Evol. 2025 Sep 25;15(10):e72179. doi: 10.1002/ece3.72179 (PMC12461107; doi:10.1002/ece3.72179)

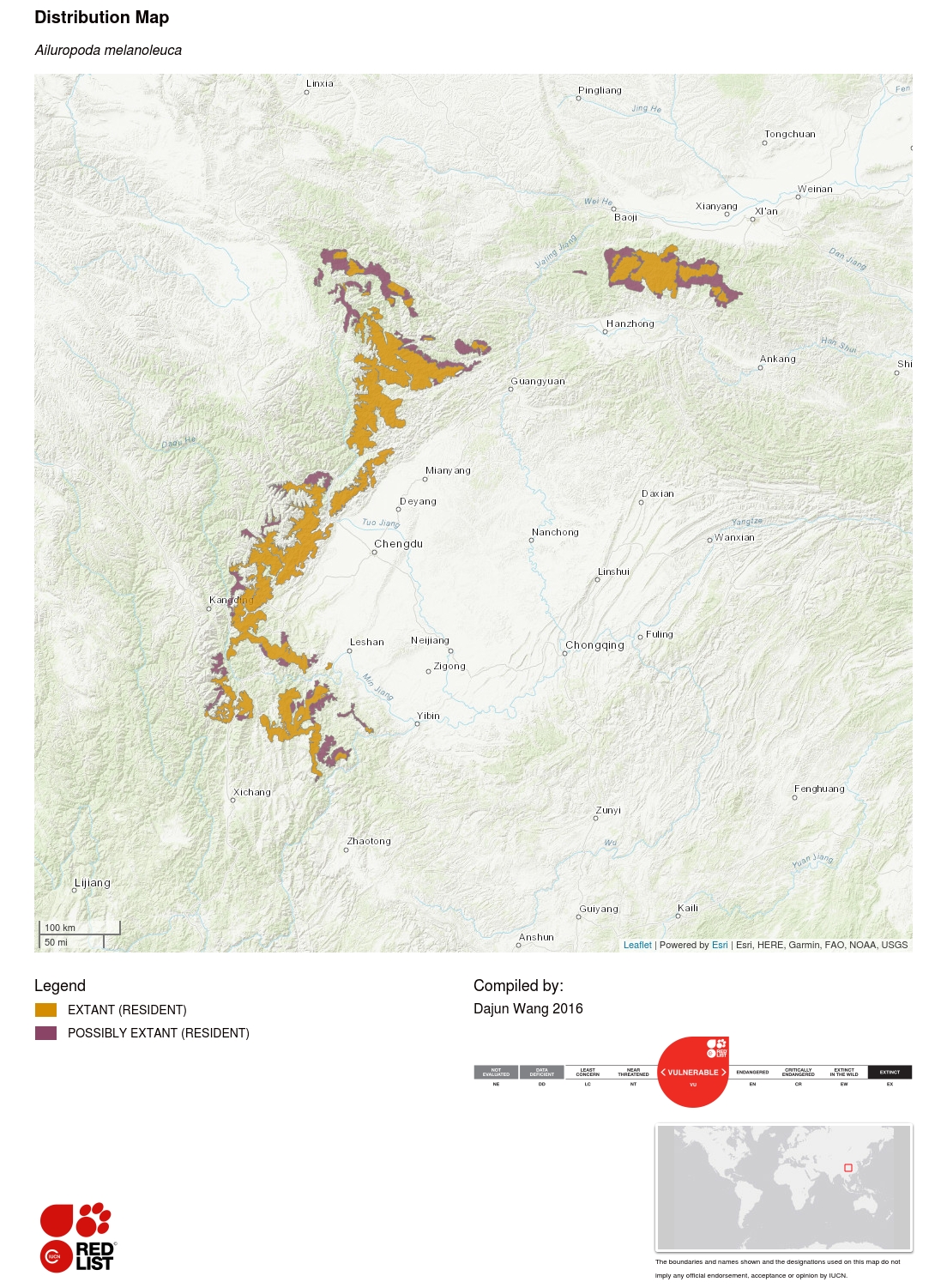

Supplement: Supplementary file 1 — Figure S1: Geographic distribution of the giant panda ( Ailuropoda melanoleuca ; IUCN 2024). [file ECE3-15-e72179-s005.tif]

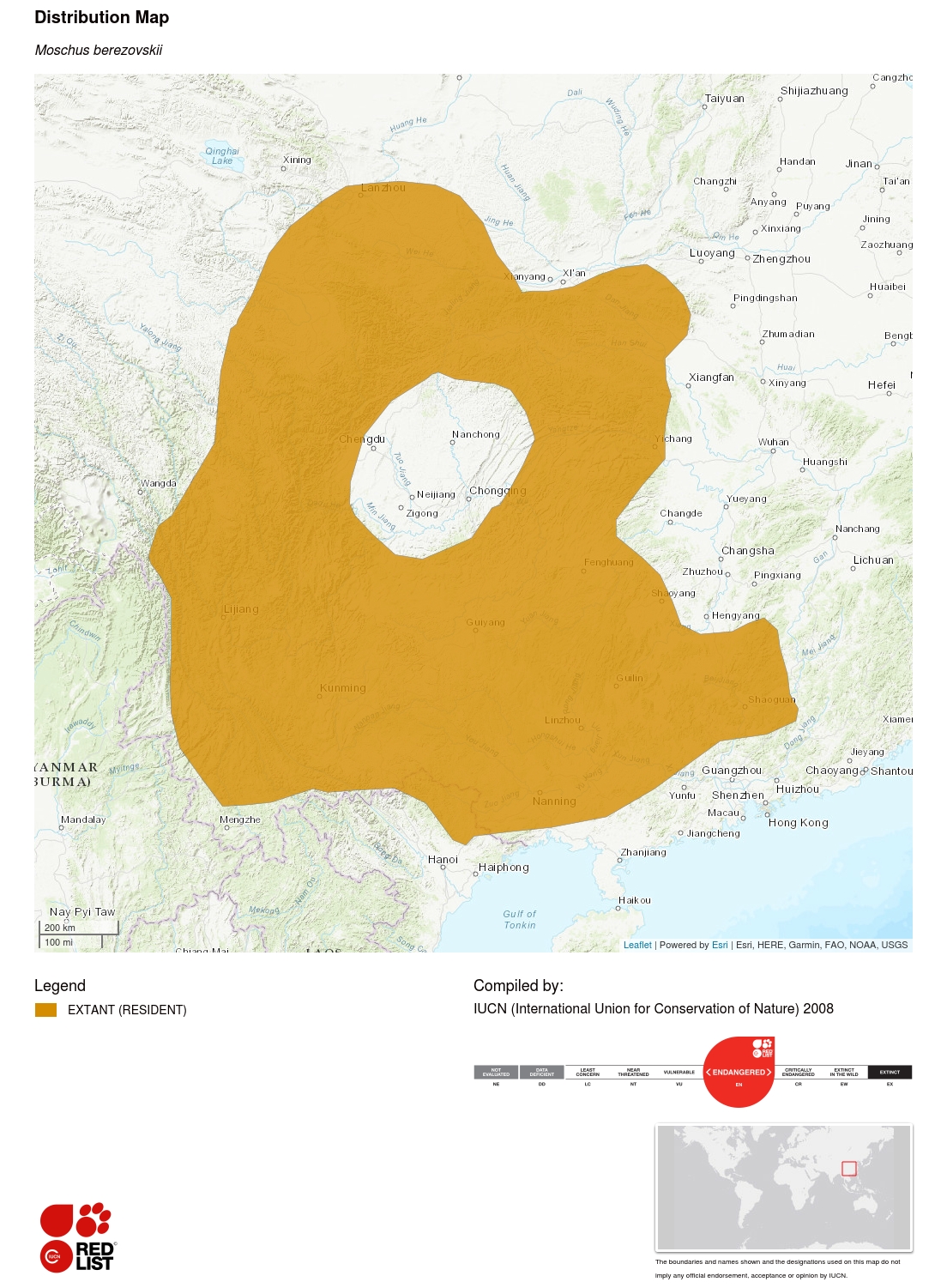

Supplement: Supplementary file 2 — Figure S2: Geographic distribution of the forest musk deer ( Moschus berezovskii ; IUCN 2024). [file ECE3-15-e72179-s003.tif]

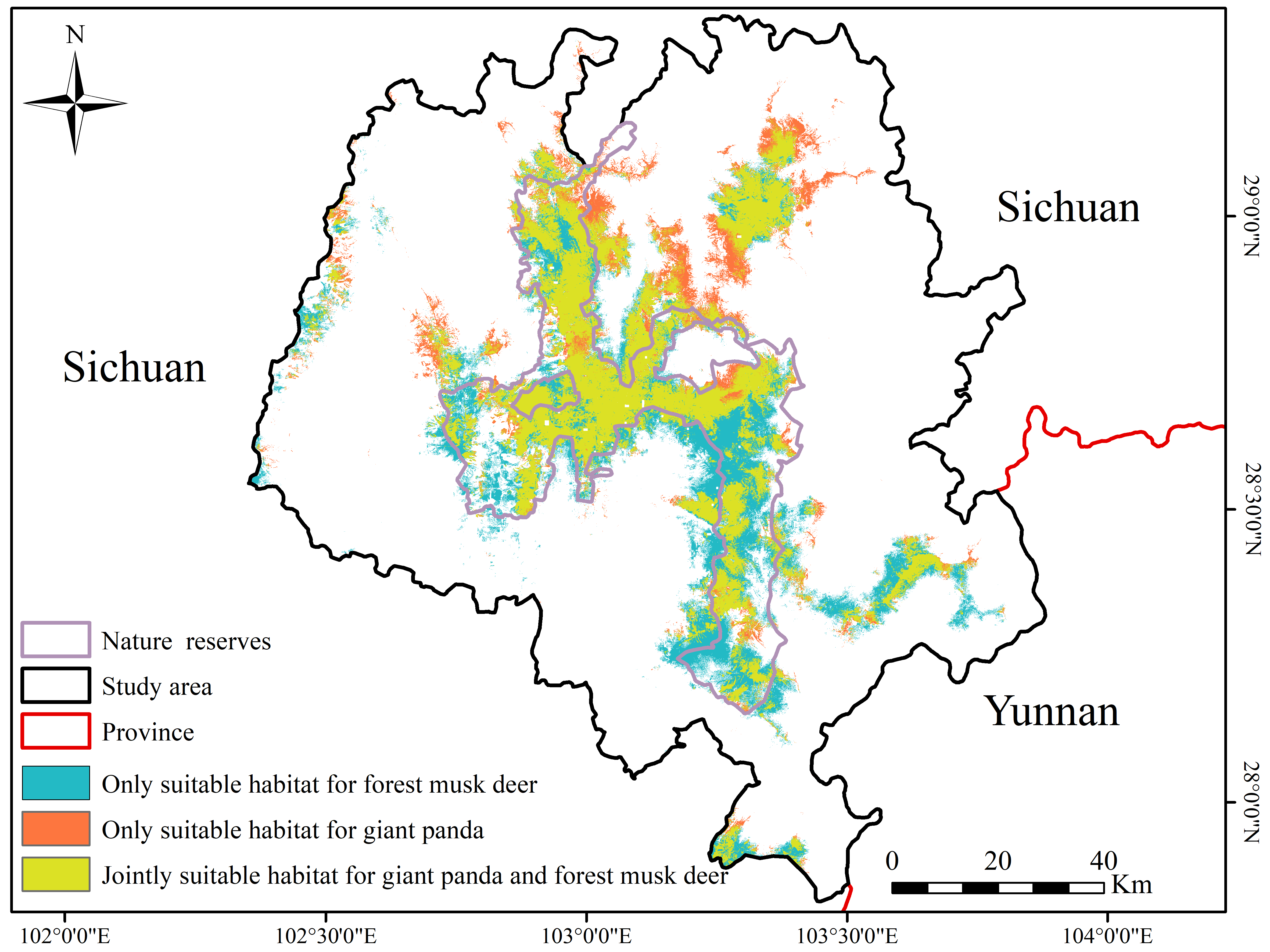

Supplement: Supplementary file 3 — Figure S3: Potential suitable habitats for giant panda and forest musk deer under the current period. The suitable habitat area solely for forest musk deer is 1099.84 km2, while that for giant panda is 364.39 km2, and the jointly suitable habitat area for both species is 1644.96 km2. [file ECE3-15-e72179-s004.tif]
