# Supplementary material for: Assessing the Giant Panda Protected Areas and Habitat Trends for Sympatric Endangered Species: A Climate Change Perspective
Source: Ecol Evol. 2025 Sep 25;15(10):e72179. doi: 10.1002/ece3.72179 (PMC12461107; doi:10.1002/ece3.72179)
Supplement: Supplementary file 4 — Appendix S1: Selection of feature combination and regularization multiplier for optimizing MaxEnt model complexity of giant pandas. [file ECE3-15-e72179-s001.docx]

Appendix A. Selection of feature combination and regularization multiplier for optimizing MaxEnt model complexity of giant pandas

| fc | rm | AICc |
| --- | --- | --- |
| LQHP | 0.5 | 6964.249 |
| LQHP | 0.5 | 6964.249 |
| L | 4 | 6891.331 |
| L | 3.5 | 6891.209 |
| L | 2 | 6887.272 |
| L | 2.5 | 6887.063 |
| L | 3 | 6886.743 |
| L | 1.5 | 6885.416 |
| L | 1 | 6883.9 |
| L | 0.5 | 6879.506 |
| LQ | 4 | 6877.255 |
| LQ | 3.5 | 6875.702 |
| LQ | 3 | 6874.258 |
| LQ | 2.5 | 6862.509 |
| H | 0.5 | 6859.764 |
| LQH | 0.5 | 6852.413 |
| LQ | 2 | 6847.081 |
| LQ | 1.5 | 6835.847 |
| H | 1 | 6824.509 |
| LQ | 1 | 6814.613 |
| H | 4 | 6812.306 |
| LQHP | 1 | 6810.853 |
| LQHP | 1 | 6810.853 |
| H | 3.5 | 6808.909 |
| H | 3 | 6806.27 |
| LQH | 4 | 6799.186 |
| H | 2.5 | 6798.898 |
| LQH | 3.5 | 6797.589 |
| LQH | 3 | 6794.255 |
| LQH | 2.5 | 6793.983 |
| H | 2 | 6788.595 |
| H | 1.5 | 6787.784 |
| LQHPT | 1.5 | 6787.19 |
| LQHP | 1.5 | 6782.799 |
| LQHP | 1.5 | 6782.799 |
| LQHPT | 2 | 6782.743 |
| LQH | 2 | 6779.804 |
| LQH | 1 | 6775.846 |
| LQHP | 2 | 6774.889 |
| LQHP | 2 | 6774.889 |
| LQH | 1.5 | 6771.837 |
| LQ | 0.5 | 6769.589 |
