# Supplementary material for: Assessing the Giant Panda Protected Areas and Habitat Trends for Sympatric Endangered Species: A Climate Change Perspective
Source: Ecol Evol. 2025 Sep 25;15(10):e72179. doi: 10.1002/ece3.72179 (PMC12461107; doi:10.1002/ece3.72179)
Supplement: Supplementary file 5 — Appendix S2: Selection of feature combination and regularization multiplier for optimizing MaxEnt model complexity of forest musk deer. [file ECE3-15-e72179-s002.docx]

Appendix B. Selection of feature combination and regularization multiplier for optimizing MaxEnt model complexity of forest musk deer

| fc | rm | AICc |
| --- | --- | --- |
| LQH | 0.5 | NA |
| H | 0.5 | NA |
| LQHP | 0.5 | NA |
| LQHP | 1 | 2167.084 |
| H | 1 | 2144.309 |
| LQH | 1 | 1998.094 |
| H | 1.5 | 1986.69 |
| H | 2 | 1955.578 |
| H | 2.5 | 1952.763 |
| H | 3 | 1944.314 |
| LQH | 1.5 | 1941.776 |
| H | 3.5 | 1937.16 |
| LQ | 0.5 | 1934.915 |
| H | 4 | 1933.677 |
| LQHP | 1.5 | 1931.307 |
| LQH | 2 | 1916.91 |
| LQHP | 3 | 1913.756 |
| L | 4 | 1913.559 |
| LQH | 2.5 | 1911.902 |
| L | 3.5 | 1910.105 |
| LQHP | 2.5 | 1909.562 |
| LQH | 3 | 1908.266 |
| LQHP | 4 | 1907.966 |
| L | 2.5 | 1907.348 |
| L | 3 | 1906.827 |
| LQHP | 2 | 1905.983 |
| LQ | 3 | 1904.274 |
| L | 2 | 1904.195 |
| L | 0.5 | 1903.857 |
| LQH | 4 | 1903.759 |
| LQ | 4 | 1903.759 |
| LQHP | 3.5 | 1903.333 |
| LQ | 2.5 | 1901.349 |
| L | 1.5 | 1900.888 |
| LQH | 3.5 | 1899.903 |
| LQ | 3.5 | 1899.903 |
| LQ | 2 | 1898.789 |
| L | 1 | 1898.051 |
| LQ | 1 | 1897.221 |
| LQ | 1.5 | 1896.564 |
